# Human Listeriosis Presenting as Breast Abscess: Report of a Rare Case

**DOI:** 10.7759/cureus.1006

**Published:** 2017-02-01

**Authors:** Venkataramana Kandi

**Affiliations:** 1 Department of Microbiology, Prathima Institute of Medical Sciences

**Keywords:** listeria sp, breast abscess, human pathogen, gram positive bacilli, normal intestinal flora of animals

## Abstract

An abscess is defined as a collection of pus in various tissues of the body including skin and other organs. Abscesses most commonly are formed on the skin under the armpits, groin areas, and rectal areas. Most abscesses involve microbial infections with few remaining sterile. The treatment of abscesses includes both medical and surgical intervention. In the era of multidrug resistance, isolation and identification of the causative microbe and testing for antimicrobial susceptible patterns assume greater significance for the better management of patients, thereby reducing the resultant morbidity and mortality. *Listeria* spp. are a group of aerobic and non-spore forming gram-positive bacilli. They are present in the environment, soil, and water. *Listeria* spp. have also been noted to be present as a normal intestinal flora of animals. They are known for their ability to thrive under both cold and hot environmental conditions. Human infections with *Listeria* spp. have not been frequently reported, mostly because of the difficulty in laboratory identification and complex clinical presentations. In humans, *Listeria* spp. have been frequently responsible for food poisoning and neonatal meningitis. Although not considered as a classic pathogen, *Listeria* spp. are associated with infections in elderly people, pregnant women, newborns, and persons with weakened immune systems. This report presents a case of breast abscess caused by *Listeria* spp. in a young lactating female belonging to rural India.

## Introduction

An abscess is a small area of skin (dermis and deeper layers of skin), which is inflamed and infected. Abscesses can also occur in any organ of the human body (liver, lung, kidneys, and brain). The etiology of abscesses varies greatly with most being caused by either or both aerobic and anaerobic bacteria and rarely caused by parasites, fungi, and foreign bodies. The bacterial species, which are most frequently associated with abscesses, include *Staphylococcus aureus*, *Streptococcus *sp., and other coagulase negative *Staphylococci*. Gram-negative bacteria including *Escherichia coli*, *Klebsiella *sp., *Proteus *sp., *Pseudomonas *sp., *Pasteurella *sp., and others have also been infrequently associated with abscesses. Most abscesses are superficial and need no medical treatment. Complications with abscesses include dissemination and development of sepsis, usually among immunocompromised and debilitated patients. Abscesses of late have also been reported to be caused by rare bacterial species; therefore, early identification of abscesses, their etiology, and antimicrobial susceptibility patterns assume greater significance for initiation of appropriate therapeutic measures. Predisposing factors for the formation of abscesses include chronic alcoholism, intravenous drug abuse, chronic treatment with steroids, patients on cancer chemotherapy, patients suffering from leukemia, peripheral vascular diseases, diabetes, acquired immunodeficiency syndrome (AIDS), and Crohn's disease. Other risk factors that can contribute to abscesses include poor hygiene, severe burns, septicemia, and surgical procedures.

*Listeria *spp. are a group of aerobic and non-spore forming gram-positive bacilli. They are present in the environment, soil, and water.* Listeria *spp. have also been noted to be present as a normal intestinal flora of animals. They are known for their ability to thrive under both cold and hot environmental conditions. Human infections with *Listeria *spp.* *have not been frequently reported, mostly because of the difficulty in laboratory identification and complex clinical presentations. In humans, *Listeria *spp.* *have been frequently responsible for food poisoning and neonatal meningitis. Although not considered as a classic pathogen, *Listeria *spp.* *are associated with infections in elderly people, pregnant women, newborns, and persons with weakened immune systems. *Listeria *spp. are ubiquitous in the environment and are commensal members of the gut flora in many mammals [1-3]. It has the ability to survive in extreme environmental conditions including extreme pH, high salt concentration, dry environments, and hot as well as under refrigeration temperatures. Although *L monocytogenes *was recognized as an animal pathogen over 80 years ago, the first outbreak confirming an indirect transmission from animals to humans was reported only in 1983 in Canada’s Maritime Provinces. In that outbreak, cabbages, stored in the cold over the winter, were contaminated with *Listeria *spp. through exposure to infected sheep manure. A subsequent outbreak in California in 1985 confirmed the role of food in disseminating listeriosis. Since then *Listeria *species have been implicated in many outbreaks of food-borne illness, most commonly from exposure to contaminated dairy products and prepared meat products [4].

*Listeria *spp. are a group of facultative intracellular bacteria, which pose a potential public health problem related to consumption of contaminated food. Human listeriosis is clinically classified as perinatal listeriosis, neonatal listeriosis, and adult listeriosis. Human infections caused by *Listeria *spp. present typically as meningitis, bacteremia, and gastroenteritis [5]. Currently there are 17 correctly identified species of *Listeria *including *L monocytogenes*, *L ivanovii*,* L seeligeri*, *L innocua*,* L welshimeri*, *L grayi*, *L* *fleischmannii*, *L marthii*, *L rocourtiae*,* L floridensis*,* L aquatica*, *L newyorkensis*,* L grandensis*, *L riparia*, *L cornellensis*, *L booriae*,and *L weihenstephanensis*. Among these species only six are associated with human beings, with *L monocytogenes* and *L ivanovii* as most frequent causes of infections in humans [6]. *Listeria *spp. may morphologically resemble *Corynebacteria*, *Streptococci, *or *Pneumococci *on direct smears and hence there is a problem of misidentification and also the chance of disregarding it as a non-pathogen or a contaminant. This report presents a case of breast abscess caused by *Listeria *spp. in a young lactating patient in India. Informed consent was obtained from the patient for this study. PIMS-IEC granted approval for this study and the approval number is PIMS/IEC/2016-18790. The approval confirms that all needed care and ethical concerns were addressed as specified by the institutional ethical committee while handling the animal subjects involved in the study.

## Case presentation

A 21-year-old lactating female presented to a hospital with complaints of a lump in her right breast. On clinical examination, tender, painful, and erythematous swelling was found with a diameter of 3 cm × 1 cm. The patient’s history revealed that the lump had started to grow in size gradually for four months and was initially firm, painless, and later turned out to be painful and tender. There was no history of trauma, fever, nausea, vomiting, and the patient had no underlying debilitating conditions. She was afebrile on admission, and all the vital signs were within normal limits. There was no spontaneous discharge from the abscess and no lymphadenopathy. The patient gave no history of recent medication. A history of working in a dairy farm as a part of living was noted.

An incision and drainage of the abscess was performed under sterile conditions and by using a freezing spray. Since the patient’s condition was stable, no antibiotic was prescribed, and a decision to observe the patient closely until the culture report arrived was taken.

Gram stain of the aspirated pus revealed short gram-positive bacilli that were arranged randomly. The culture on blood agar grew 0.5–1 mm in diameter—small, round, translucent, and ß-hemolytic colonies—and there was no growth on MacConkey’s agar. Gram staining of the isolated colonies revealed very short gram-positive bacilli arranged in pairs and randomly. They were found to be nonspore-forming, non-branching, and non-capsulated. Conventional biochemical reactions showed that the isolated bacterium was catalase positive (differentiating it from morphologically resembling *Streptococci*) and negative for oxidase (production of cytochrome oxidase enzyme) and nitrate reduction test (inability to reduce nitrate to nitrite). Tests for indole (production of tryptophanase), hydrogen sulfide (H_2_S) production, urease production, citrate utilization, and gelatin hydrolysis were negative. The isolated bacterium was found to be positive for Voges-Proskauer test (produced acetoin from the fermentation of glucose through the butanediol pathway), produced only acid from glucose, and did not ferment other sugars including mannitol, mannose, lactose, sucrose, and xylose.

The isolated bacterium was motile at 25°C showing tumbling motility and non-motile at 37°C. Cold enrichment at 4°C after a prolonged incubation of more than three days was positive. Other laboratory tests including growth in the presence of 10% sodium chloride (NaCl) (salt tolerance test) and Christie-Atkins-Munch-Petersen (CAMP) test were positive. The culture on bile esculin agar revealed the growth of black colored colonies after an incubation of 48 hours at 35°C. A histopathological study of the tissue biopsy revealed extensive inflammation with no signs of fibromatosis and granuloma. Sereny test, an experimental animal inoculation test, was performed to demonstrate the invasive property of the isolated strain. With the help of a sterile cotton swab, the culture broth of isolated *Listeria *spp. was applied on a guinea pig eye. The inflammation of the eye (keratoconjunctivitis) was noted after 48 hours, confirming its virulence.

The isolated bacterium was identified as belonging to *Listeria *spp. with the available conventional laboratory tests [5]. Antimicrobial susceptibility testing of the isolated bacterium was carried out using the Kirby-Bauer disk diffusion method. The isolate was found sensitive to most tested antibiotics including amikacin, ciprofloxacin, ofloxacin, amoxicillin-clavulanic acid, trimethoprim-sulfamethoxazole, ceftriaxone, ceftazidime, piperacillin-tazobactam, imipenem, and meropenem. Resistance to penicillin and ampicillin was noted.

Empirical therapy including amoxicillin-clavulanic acid 1.2 mg three times a day (TID) and 400 mg ciprofloxacin TID was started. The patient responded well and had an uneventful recovery.

## Discussion

*Listeria *has a worldwide distribution and human listeriosis is seen in both immunocompromised and immunocompetent conditions [3]. Human listeriosis is a zoonotic disease and usually presents as food poisoning, bacteremia, or meningitis. Other infections attributed to *Listeria *spp. include endocarditis, myocarditis, arteritis, pneumonia, pleuritis, cholecystitis, peritonitis, arthritis, osteomyelitis, sinusitis, otitis, conjunctivitis, and ophthalmitis, and in a pregnant woman, abortion, stillbirth, and premature birth may be seen [3]. A case of breast implant infection with *L monocytogenes *has been reported in a young and healthy female, interestingly with no history of exposure to farm or exotic animals [7]. In the present case, history of close proximity with animals can be the cause of the breast abscess.

Regarding the treatment of invasive listeriosis and considering the fact that *Listeria *species are intrinsically resistant to broad-spectrum cephalosporin antibiotics, ampicillin and its derivatives could be considered as preferred agents [8]. In the present case also, the patient responded well to a combination therapy including amoxicillin-clavulanic acid and ciprofloxacin.

Due to its morphological resemblance with other *Corynebacterium *species (diphtheroids),* L monocytogenes* is often regarded as a skin commensal or contaminant in the laboratory. *Listeria *spp. also resemble other less significant gram-positive bacteria occasionally causing human infections including *Arcanobacterium *sp, *Erysipelothrix rhusiopathiae*, *Rothia *sp., *Rhodococcus *sp., *Trueperella *sp., *Aureobacterium *sp., *Bifidobacterium *sp., *Brevibacterium *sp., *Cellulomonas *sp., *Dermabacter *sp., *Arcanobacterium*, and other aerobic and anaerobic bacteria. It is difficult to isolate this organism from certain clinical specimens, particularly from tissues obtained by surgery.

Pathogenicity of the *Listeria *spp. could be identified by testing for the presence of a virulence factor named Listeria adhesion protein B (LapB), as noted by a recent research report [9].

Laboratory methods for identification of *Listeria *of late have undergone great improvement with the availability of advanced and molecular techniques. Automated identification systems like MicroScan WalkAway (Dade Behring Inc., CA, USA), API 20 E (bioMerieux Inc., NC, USA), and Vitek ID-GP (bioMérieux, France), although available, are usually confined to few centers due to cost constraints. Matrix-assisted laser desorption/ionization time-of-flight (MALDI-TOF) mass spectroscopy, nucleic acid amplification tests (NAATs), including the polymerase chain reaction (PCR), fluorescent amplified fragment length polymorphism (AFLP), and pulsed field gel electrophoresis (PFGE) can be employed for species identification and confirmation of *Listeria *spp. [10].

## Conclusions

Although *Listeria *spp. are considered as present normally in the environment and in the animals, there is a potential risk of serious infections in humans with underlying debilitating conditions. Human infections with *Listeria *spp. remain under reported due to its unusual clinical presentation and difficult laboratory identification owing to its complex physiological and biochemical characters. Clinical microbiologists should be proactive in the laboratory identification of *Listeria *spp. as most isolates of *Listeria *spp. are misidentified as other morphologically similar bacteria and are frequently ignored as laboratory contaminants. Identification of susceptible population, appropriate clinical suspicion, and improved laboratory identification methods will certainly contribute to increased identification of *Listeria *spp. in human clinical specimens and thereby reduce the morbidity due to human listeriosis.
